# An Efficient Middle Layer Platform for Medical Imaging Archives

**DOI:** 10.1155/2018/3984061

**Published:** 2018-06-21

**Authors:** Atilla Ergüzen, Erdal Erdal

**Affiliations:** Department of Computer Engineering, Kırıkkale University, 71450 Kırıkkale, Turkey

## Abstract

Digital medical image usage is common in health services and clinics. These data have a vital importance for diagnosis and treatment; therefore, preservation, protection, and archiving of these data are a challenge. Rapidly growing file sizes differentiated data formats and increasing number of files constitute big data, which traditional systems do not have the capability to process and store these data. This study investigates an efficient middle layer platform based on Hadoop and MongoDB architecture using the state-of-the-art technologies in the literature. We have developed this system to improve the medical image compression method that we have developed before to create a middle layer platform that performs data compression and archiving operations. With this study, a platform using MapReduce programming model on Hadoop has been developed that can be scalable. MongoDB, a NoSQL database, has been used to satisfy performance requirements of the platform. A four-node Hadoop cluster has been built to evaluate the developed platform and execute distributed MapReduce algorithms. The actual patient medical images have been used to validate the performance of the platform. The processing of test images takes 15,599 seconds on a single node, but on the developed platform, this takes 8,153 seconds. Moreover, due to the medical imaging processing package used in the proposed method, the compression ratio values produced for the non-ROI image are between 92.12% and 97.84%. In conclusion, the proposed platform provides a cloud-based integrated solution to the medical image archiving problem.

## 1. Introduction

Picture archiving and communication systems (PACS) and hospital information management systems (HIMS) have provided great improvements in the field of health services. However, these systems have brought along some problems as well as the advantages of development. The amount of data is increasing day by day that has a vital role in diagnosis and treatment. Managing, querying, analyzing, and backing up these massive data with traditional methods are a major problem for hospitals. Recently, researchers have shown an increased interest in this area. Turkey is among the developing countries in the healthcare and modern health services, which are becoming widespread. The average rate of magnetic resonance imaging (MRI) scan for the Organization for Economic Co-operation and Development (OECD) countries is 52 per thousand people per year. Turkey is the first among these countries with 119 MRI scans per thousand people per year [1]. Moreover, according to the World Population Census report of the United Nations (UN), it is predicted that Turkey, whose current population is 78 million 666 thousand, will be 87 million 717 thousand in 2030 and 95 million 819 thousand in 2050 [2]. Despite this increase in the community, it is estimated by the Turkish Statistical Institute (TUIK) that Turkey will attend the “elderly countries” category within 15–20 years at the latest due to the declining fertility rate [3]. This problem is a growing public health concern worldwide. The evidence presented thus far supports the idea that it is mandatory to process and manage big data.

The term big data encompasses (a) massive amount of data (volume), (b) amount of increase in data (velocity), (c) kind of data (variety), and (d) noise, biases, and abnormality of data (veracity). Storing and analyzing big data on a regular basis is a challenge which is a hot topic in the literature [4]. The term medical image refers to big data characterized by volume, velocity, variety, and veracity. Managing medical images big data (MIBD) with traditional methods is a complicated problem for all medical centers.

Traditional methods must be interpreted with caution because of increase in (1) the time of access to the data, (2) length of time patients spend in the hospital, (3) requirement of additional clinical and laboratory examination, and (4) storage costs [5]. For these reasons, HIMS, PACS, and archive systems must be adapted to big data structures. These new structures provide improved, cost-effective, and quick access management.

The Apache Hadoop Foundation, working on Linux clusters with the latest developments, has developed a distributed software framework for data warehouses at petabyte scale [6]. This framework uses the MapReduce programming model and reduces the fault tolerance by storing the data in parallel on the cluster. A wide variety of researchers and organizations are using Hadoop technology in the field of healthcare [6].

Also, with the development of big data technologies, alternative database systems have been developed instead of relational database management systems (RDBMS). These databases are known as Not Only SQL (NoSQL) databases. MongoDB is a document-based flexible and unstructured NoSQL database that stores all objects as serialized JavaScript Object Notation (JSON) and Binary JSON (BSON). It has a feature that supports automatic sharding and stores the data on available servers by dividing them into partitions. This feature creates a dynamically balanced load without requiring any intervention. The maximum document size that can be stored in MongoDB is limited to 16 MB, but large data can also be stored via the GridFS built-in structure [7, 8].

In this study, a MIBD-processing and MIBD-archiving middle layer platform based on Hadoop and MongoDB architecture has been developed. The developed platform consists of a four-node Hadoop cluster, one name node and three data nodes. Distributed MapReduce algorithm and MongoDB are executed on each node. The platform consists of four primary stages. First, the medical image package contains one or more images. Every image in this package is sent to the available server in the Hadoop cluster for parallel processing. All processes are executed on the Hadoop cluster with the MapReduce programming model. In the second step, raw data are processed and compressed using a medical image processing package (MIPP). This packet divides the medical images into the region of interest (ROI) and the remaining region (non-ROI) by the region-based active contour method (RBACM). The ROI region is compressed with the JPEG-LS lossless compression algorithm. Then, optical character recognition (OCR) algorithm is applied to the non-ROI region. When the patient information is extracted, the unnecessary region in the image (non-ROI) is deleted. Using the Huffman coding algorithm, OCR process result data are compressed. At the last stage of this package, all data are consolidated; finally, a file is generated according to the specially designed dynamic file structure. Then the obtained data and corresponding keys are stored in MongoDB. The last feature on the platform is a search engine with a user interface. The developed search engine is designed to search for the patient ID (HIMS unique ID) or the defined criteria such as citizenship identification number, name, and surname. After the search is done, all stages in the system are reversed, and the image is delivered to the user as original DICOM object.

**Figure 1:**
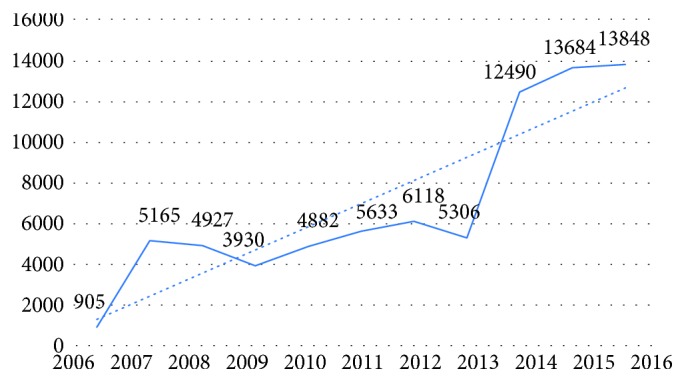
The number of MRI by years.

Sample MRI images that are obtained from Kırıkkale University Faculty of Medicine have been used to evaluate the study performance and validity. There are 76,888 MRI images of the institution between the years 2006 and 2016. Since the PACS has not been used until 2013, the data in this time interval have been ignored. Therefore, 51,446 MRI images have been archived between the years 2013 and 2017 as shown in Figure 1.

**Figure 2:**
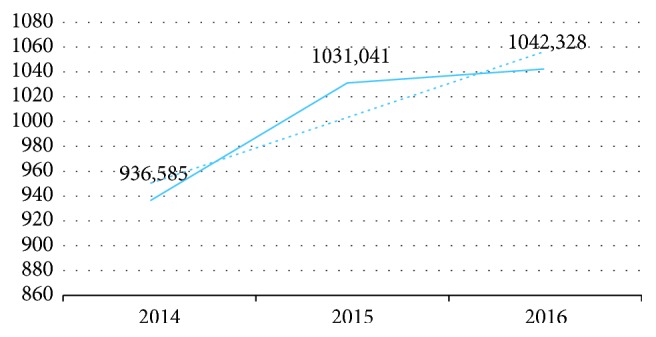
Data amounts by years.

The last three years' data are presented in Figure 2. These data are stored in three different centers, and this backup and recovery storages increases the data size. The main pillars of the study are (i) to decrease the image size with compression, (ii) to reduce the cost of storage, (iii) to provide a backup approach service-based system integration, (iv) to enable an easy, fast, and detailed search, and (v) to establish a flexible, scalable, and modern big data system.

The utilization of MapReduce/Hadoop and MongoDB is increasing in the literature. However, a complete service-based medical image archive platform has not been unprecedented.

Our motivation for developing this method is the deficiencies in the literature: (i) medical image processing in a distributed platform before storage process, (ii) a well-defined search engine for the developed archive system, and (iii) store data in an efficient database with a custom-defined file structure.

The following contributions have been presented in this study. (1) A distributed platform has been built using MapReduce/Hadoop architecture. Thanks to this structure, medical images are processed quickly and efficiently. (2) The medical image processing package (MIPP) has been applied to medical images to produce a more efficient structure with higher compression ratios. (3) All data have been stored in a stable, consistent, and dynamic file structure. (4) These data are stored in MongoDB, which is powerful, scalable, flexible, and useful. (5) The developed search engine provides fast, efficient, and precise results. This study aims to bridging the gap in this area in the literature.

## 2. Background

In this section, background studies of implemented algorithms and methods are presented.

### 2.1. Medical Image Processing Package

In this study, a preprocessing operation has been applied to medical images. The steps of the operation are as follows:

First, partitioning has a significant role in image analysis to separate some pixels or multiple regions. This process also identifies the position and boundaries of the defined regions in the picture. Most studies in this field have only focused on this aim. However, the active contour model is preferred due to its successful properties such as region growth, threshold, and edge detection [9, 10]. The region-based active contour method is suitable for this model that is used for the separation of the region of interest (ROI) and the remaining part (non-ROI) in the medical image [11, 12]. Because, this method is more sensitive to noise and has better performance; therefore, it is often used to distinguish the region of interest in medical images [13–17].

Second, the OCR can be defined as the machine equivalent of human readout, which provides an editable translation of the text on the images. The OCR method is applied to the non-ROI region of the medical image, and the metadata and header information in the picture are obtained. In the literature, the term OCR, which researchers are trying to develop to increase the success of the algorithm, tends to be used to refer to extract and identify characters [18].

The JPEG-LS algorithm is an easy-to-implement lossless image compression algorithm [19]. It has excellent computational efficiency and coding features. The ROI part of the medical image is a critical region that is not tolerant of any data loss. For this reason, a lossless compression algorithm has been applied to this area. JPEG-LS is preferred against other lossless compression algorithms due to (i) easy applicability, (ii) low complexity, and (iii) better performance [19]. This algorithm is still being studied, and it is frequently used on medical images [20].

Finally, Huffman coding, which is an entropy coding algorithm in information theory and computer science, is widely used for lossless data compression [21]. The Huffman coding algorithm uses a statistical structure depending on the frequency of the data. The coding mechanism is mainly based on the incidence of the alphabet in the dataset. The fundamental principle of this structure is to represent the more commonly used data with fewer bits. It is easy to code and requires little slight input/output (I/O) operation. In this study, Huffman coding algorithm is applied to the data obtained by OCR process. This method, which is at the core of many compression algorithms, is aimed to produce higher compression ratio.

### 2.2. Hadoop/MapReduce

Hadoop/MapReduce, one of the hot topics in big data literature, is a data processing and analysis technology that revolutionizes the field of computer science. MapReduce is a Google-developed solution for handling large amounts of data, and the primary objective is indexing of billions of web pages [22]. MapReduce is a two-level parallel data processing model developed with Java: Map and Reduce. The data itself are subdivided into small pieces, and MapReduce is used to distribute computations to the place where it is located (Map) and to sum up the results (Reduce). Hadoop is an open source solution consisting of MapReduce and the Hadoop Distributed File System (HDFS) [23, 24]. Hadoop is a distributed architectural platform that comprises a name node and many data nodes. In recent years, this technology has frequently been used in the field of health services (i) to develop the framework, (ii) to develop medical large data processing systems, and (iii) to analyze large-scale medical images [6, 23, 24].

### 2.3. MongoDB

Relational database management systems (RDBMS) are now widely used database management systems (DBMS) nowadays. However, these methods are not effective in applications such as big data processing. For this reason, NoSQL DBMSs have been developed. Relational DBMSs are still dominant in the database market; however, NoSQL DBMS platforms and usage are increasing [25]. MongoDB, the most widely used document-based NoSQL, is an open-source DBMS. According to research by DB-ENGINES, MongoDB ranks fifth among all DBMSs, and it ranks first among NoSQL DBMS [26]. In addition, big data are featured by increased volume, high velocity, variety, and veracity. Considering these features of big data, medical images carry all the features of the big data. MongoDB provides solutions to these problems that cannot be managed by traditional software tools. MongoDB is preferred against other DBMS due to the following criteria: (i) efficient big data processing capability, (ii) most widely used document-based open-source NoSQL DBMS, (iii) convenient to be deployed in a variety of ways, and (iv) supports distributed environments with scalability feature.

MongoDB is not schema-based, and each document is stored in a BSON format. In this format, an object is an unordered name/value pair group. Since there is no table structure defined in MongoDB, any BSON format document can be inserted. Besides, MongoDB supports a distributed environment and can be deployed in a variety of ways. There are three different types of deployment: replica set, standalone, and sharded cluster [7]. In this work, sharded cluster deployment type is used for data distribution. In sharded cluster distribution, the data are—fragmented and stored on multiple servers.

GridFS is a specific MongoDB built-in function that is often used to store large files and retrieve them from a MongoDB database easily. MongoDB is used in the field of health in the literature, and satisfactory results are obtained. In this study, sharded MongoDB, in which data are distributed to four machines, is used because of its advantages and beneficial results. Moreover, the GridFS function is used because it provides an easy way to store and retrieve large files in MongoDB [7].

## 3. Materials and Methods

In this section of the study, materials and methods are presented. This part is structured into three subsections to be more detailed and conceivable. The first part is named “medical image processing and compression algorithms.” In this subsection, image segmentation processes, the process of ROI and non-ROI regions have been explained comprehensively. The second of this part is called “Infrastructure architecture.” System Architecture, Search Engine, and Reverse Preprocessing operations have been clarified in this part. Operation Parallelism subsection is the last title of this part. Hadoop/MapReduce and MongoDB and GridFS operations have been detailed in this part.

### 3.1. Medical Image Processing and Compression Algorithms

**Figure 3:**
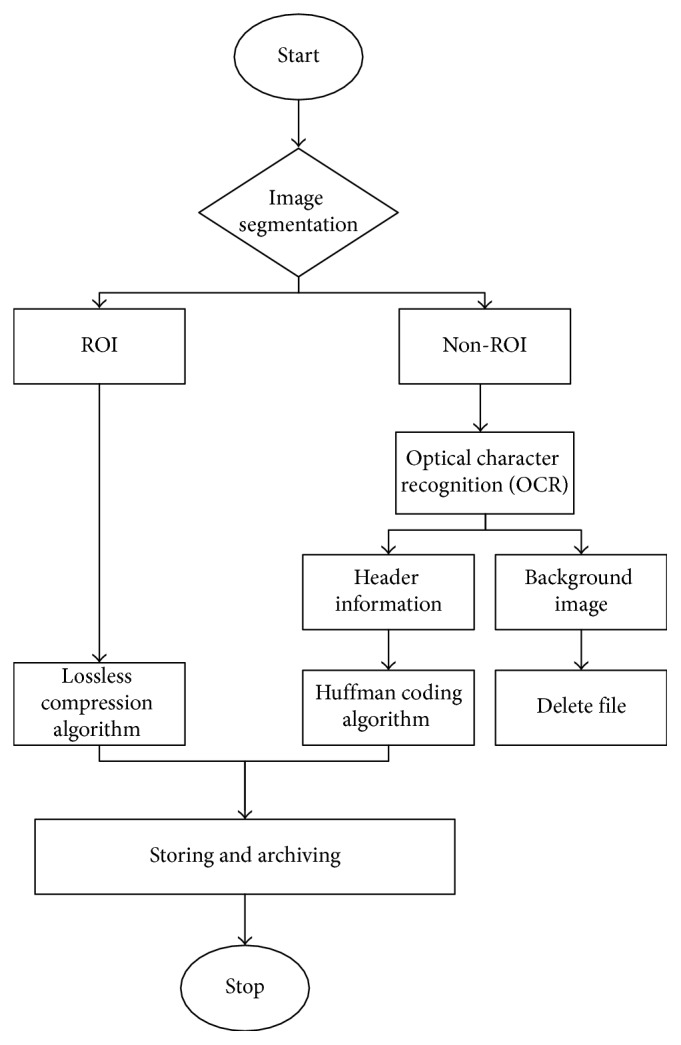
Flow chart of medical image processing package.

According to our previous work [27], each image is passed through an ROI-based preprocessing stage. This process, where the image is compressed and the raw data are generated, is categorized into four steps. The flowchart of the method is shown in Figure 3. All stages are executed on the Hadoop cluster with the MapReduce programming model.

#### 3.1.1. Image Segmentation Process

The original medical image is divided into ROI and non-ROI regions. There are many methods for this process, but the region-based active contour method, which is faster, more efficient, and higher accuracy than the other methods, is used in this study. In consequence of this process, the medical image is separated into ROI and non-ROI regions.

#### 3.1.2. The Process of ROI Region

In medical images, the area of the ROI has a vital role in the diagnosis and treatment stages, so this region does not tolerate data loss. At this point, the lossless compression algorithm is applied to compress the image. The JPEG-LS lossless compression algorithm is a well-known compression algorithm with low computational complexity and high availability. In this work, the ROI region of the image is compressed with the JPEG-LS lossless compression algorithm.

#### 3.1.3. Processes of Non-ROI Region

One of the main contributions of preprocessing is the operations applied to the non-ROI region. Following procedures are used to non-ROI region.

*(1) Optical Character Recognition*. OCR is being implemented to the non-ROI region of the medical image. By this process, meta and header information on the picture is obtained. Also, OCR operation enables to extract coordinate information, so that the original image can be reproduced. After receiving this information on the non-ROI region, the remaining area is unnecessary and ignored in the proposed method. Another key point is that other ROI-based approaches in the literature also compress the non-ROI region of the image using a lossy compression algorithm. These methods in the literature have increased the file size to be stored. However, the method utilized in this study has significantly increased the efficiency of the archiving system with ignoring the background part of the image.

*(2) Huffman Coding Algorithm*. The Huffman coding algorithm compresses the result of OCR data. The Huffman coding algorithm is a lossless compression algorithm and provides compression without loss of data. In this approach, better compression and archival results are obtained with the Huffman coding algorithm.

#### 3.1.4. Processes of Non-ROI Region

**Figure 4:**
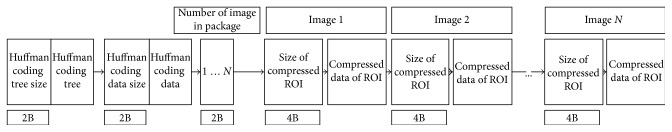
Dynamic file structure.

Another contribution of this work is developing a dynamic file structure shown in Figure 4. This file structure consists of four data pieces: (i) Huffman coding tree size and data, (ii) Huffman coding data size and data, (iii) image number, and (iv) compressed ROI size in the medical image package and data. This information of each data section is crucial to avoid irregularity among the packets. The developed file structure is suitable for medical image packages having one or more images.

### 3.2. Infrastructure Architecture

#### 3.2.1. Brief System Overview and Integration

**Figure 5:**
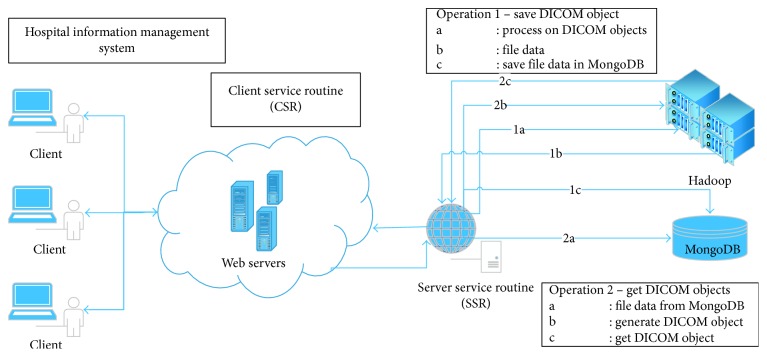
System overview.

This section of the manuscript gives a brief system overview and integration steps which are detailed in Figure 5 and addressed description.

DICOM (digital imaging and communication in medicine) is one of the most popular standards in healthcare, which makes medical image exchange easy and provides medical device independence. Therefore, all the medical images acquired from magnetic resonance imaging (MRI) devices have been stored in DICOM format. HIMS use these files for reporting, data transfer, diagnostic, and treatment purposes. Client application means a health information system which is comprehensive software developed for hospitals, deployed on a web-server having static IP.

The technical part of this study can be best treated under three headings: service routines, security issues, and Hadoop-MongoDB cluster architecture. Two different service routines have been implemented by using socket programming to achieve secure communication between client application and server application, client-service routine (CSR), and server-service routine (SSR), respectively, as shown in Figure 5. These services communicate with each other using a JSON and BSON data structure over TCP/IP. To enable the client application to connect to the server, the CSR library files have to be installed on client-side application. The CSR is responsible for sending the DICOM files to SSR and retrieving them on demand. Here, the main function of CSR is establishing a secure connection with SCR and routing health information system requests to SCR. The CSR uses two methods processed on the server, SaveFile (Operation 1) and ReadFile (Operation 2). With the first method, DICOM images and their entire metadata and the HIMSID primary key are sent to the SSR (Operation 1 in Figure 5). The ReadFile method is responsible for reading the file from the server using the primary key value (Operation 2 in Figure 5).

The server side is the most important part of this work, it is a comprehensive server platform that consists of (a) a service routine that manages the secure connection with the CSR, (b) an efficient file structure tool which splits DICOM objects in a space-saving way and can rebuild DICOM objects, and (c) Hadoop, MapReduce, and MongoDB management software tools as ad hoc developed for constituting a big data cluster.

SSR is responsible for (a) listening to the specified port number for any client application request; (b) the authentication process including primarily checking client application IP value whether this value is the same IP value belongs to registered CSR. After that process, SSR immediately checks username and password against unauthorized login; and (c) executing writing and reading operations triggered by the CSR on the cluster.

SSR stores files received DICOM by changing to its own developed format to make the archiving process fast and efficient. The SSR then rebuilds the DICOM file from own file structure and sends back when CSR sends a read request.

It is important to note that this architecture is primarily designed to make a middle layer platform between the client application and server-side Hadoop-MongoDB cluster platform. The DICOM files sent to SSR are stored in a developed file format in the Hadoop-MongoDB clusters; however, when the client requests to read that file, DICOM object is rebuilt and sent back. It does not matter that the client application is aware of where and in which format the DICOM object has been stored, the important point is that CSR should get these files in DICOM format when requested.

Another important aspect of the study is security issues of the platform. The following properties have been used for this problem:

- Client Application must have a static IP value to identify the right user accessing the SSR and to provide secured writing and readings. The IP value of CSR is stored in SSR to verify the client application that will connect to the server.
- Username and password are manually created and embedded in the system.
- JSON and BSON data structure has been used for data transfer packages which consist of HIMS ID, public key, DICOM file, and related information.
- The asymmetric encryption method has been used with private and public keys being created.

The existing literature on encryption is extensive and focuses particularly on asymmetric key encryption. Asymmetric key encryption, also known as public-key cryptography, uses two different keys, the private key, and the public key. The private key is only known to your computer; the public key is given to any computer that wants to communicate securely with your computer. To resolve the code of an encrypted message, the computer must use the public key provided by the source computer and its private key. When an encrypted message is sent from one computer to another computer, the recipient of the message cannot read the message without the private key of the message. Thanks to security measures, the security of the developed middle layer platform has been increased dramatically.

#### 3.2.2. System Architecture

**Figure 6:**
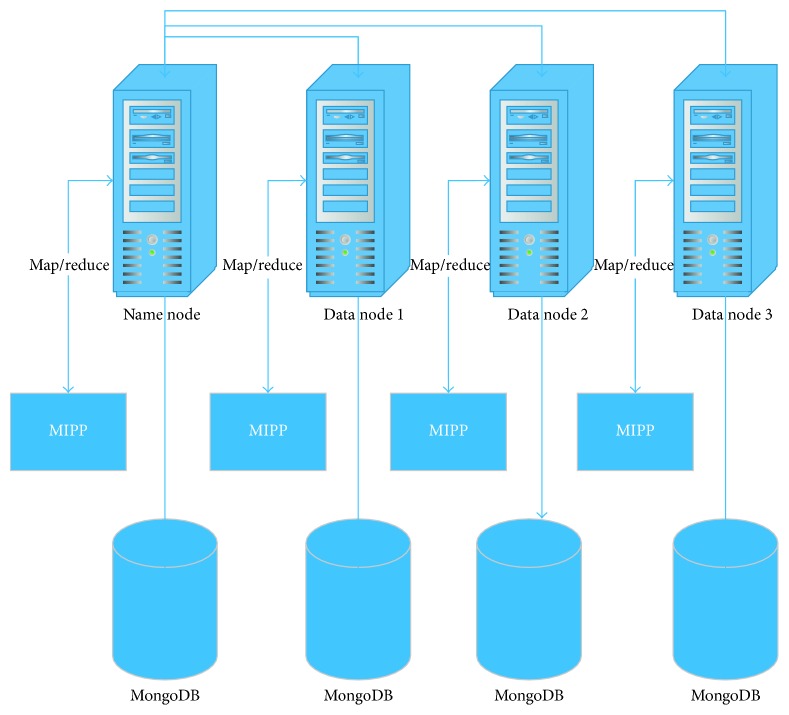
System architecture.

The proposed system consists of four-node Linux cluster: one name node and three data nodes. Redhat Enterprise Linux Server 6.0 executes on each node. Also, Java-1.6.0, MongoDB-3.4.2, and Hadoop-2.7.2 are installed on each node. In this way, each Hadoop node has a database at the same time. The designed system architecture is shown in Figure 6.

#### 3.2.3. Search Engine

A user-friendly search engine has been developed for reusage of archived files. Since the search criteria used in the improved search engine, which is defined as an index on MongoDB, a quicker and efficient search is presented. Developed system has a login page for the system security and a search engine in the search page.

**Figure 7:**
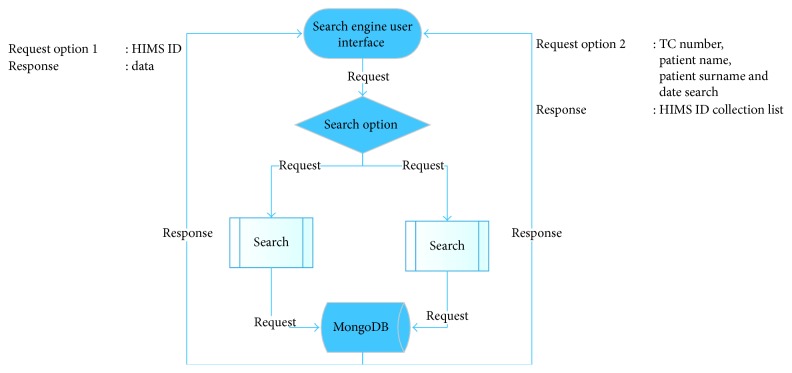
Search engine flow chart.

The flowchart showing the operation method of the search engine is presented in Figure 7. The search engine provides searching in two different ways. First one is HIMS ID; it provides an efficient and direct search by a primary index of MongoDB. The second search option is citizenship identification number, patient name, patient surname, and date. However, HIMS ID collection is listed to the user in this method.

After user selection, the first search method is used, and the data are queried with the HIMS ID. As mentioned above, the first search method returns the data package.

#### 3.2.4. Reverse Preprocessing

The result of the querying is made via the search engine, the data package received from MongoDB is processed, and the original DICOM file has been obtained. The non-ROI region of the medical image is constructed with the following steps: (1) the defined size (width, height) black background image is created; (2) the result of the OCR data which is obtained and compressed by the Huffman coding algorithm is replaced by the data coordinate; (3) by combining the obtained non-ROI region and the ROI region compressed by the lossless compression algorithm, the original medical image is obtained and presented to the user. All these steps are executed on the Hadoop cluster with the MapReduce program model, just like in the preprocessing step. When interoperability issues are considered, there is not any handicap in the developed middle layer platform. The received data are in DICOM format and are transmitted again in DICOM format, regardless of how the data are stored on the platform. In summary, the DICOM standard has not been changed.

### 3.3. Operation Parallelism

#### 3.3.1. MapReduce/Hadoop

**Figure 8:**
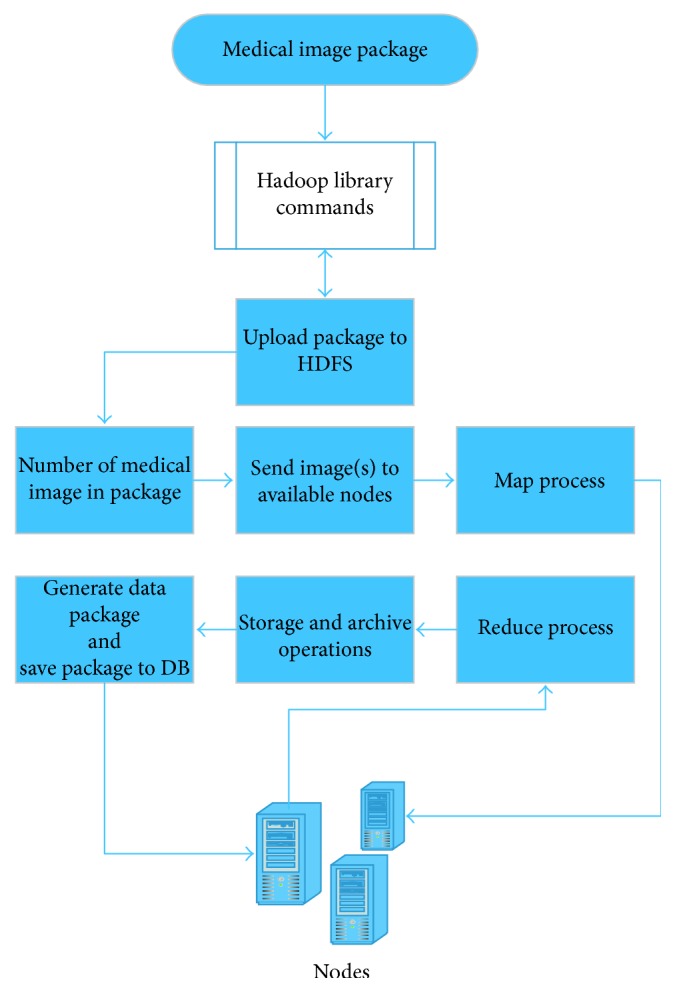
MapReduce/Hadoop steps.

In this work, Hadoop clusters are used to execute distributed MapReduce algorithms. Thanks to this cluster, a medical image processing platform is built that it is fault-tolerant and operates in parallel. The data to be stored in the archive system are loaded into HDFS via data interface, which is developed using Hadoop library commands. This cluster first determines the number of medical images contained in the uploaded package and distributes tasks to each available node. After getting the file, each node performs the Map operation, and the operations described in preprocessing part of the study. Because of this operation, the obtained data are sent to the name node that is called Reduce operation. Then all the data belonging to the package are combined and sent to the database to store. According to [28], the steps applied at this stage are shown in Figure 8.

#### 3.3.2. MongoDB and GridFS

MongoDB can share the data collections automatically with its built-in functions and store the partitioned data in available servers. In this way, a load-balanced distribution between the nodes is provided dynamically. With all these features, both capacity and throughput can be enhanced by increasing the number of nodes in MongoDB. All data are stored on four nodes, thus the platform developed with this structure is obviously fault-tolerant.

**Figure 9:**
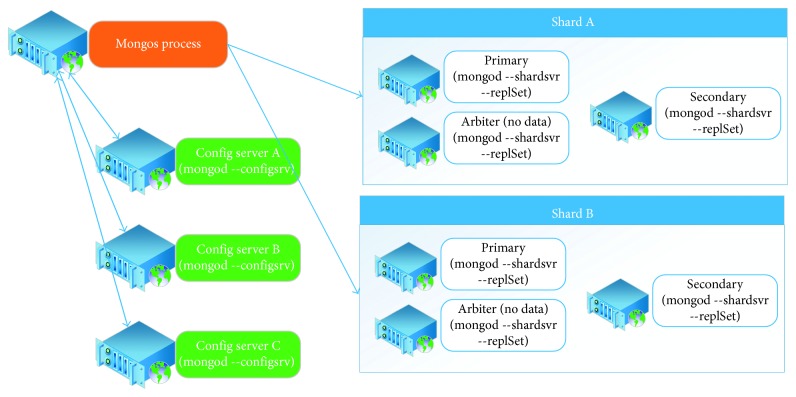
Sharded cluster method [28].

The sharded cluster method is used to provide data distribution, and an instance of this deployment is shown in Figure 9.

Database indexes are used for faster querying. In the developed system, HIMS ID value and citizenship identification number, patient name, surname, and birth date data are defined as a database index.

**Figure 10:**
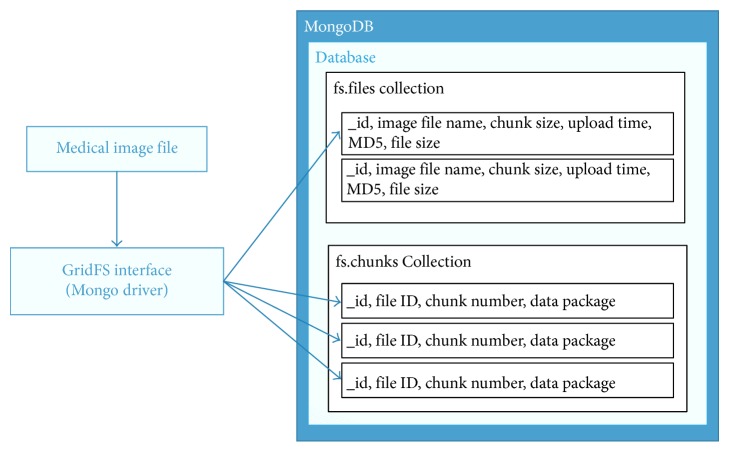
GridFS architecture.

GridFS is a MongoDB built-in file system used to manage large files. GridFS uses the replication and partition mechanism of the direct database system and does not need another storage structure. Because of storing data via GridFS, MongoDB generates fs.files and fs.chunks collections. The GridFS architecture is shown in Figure 10.

## 4. Experimental Results

### 4.1. Dataset

For the evaluation of the proposed method, the actual patient medical images have been used as shown in Figure 10. Detailed information such as the section, rate and file size of actual patient medical images used in the study are shown in Table 1.

### 4.2. Medical Image Processing and Compression Algorithm Results

The compression ratio values obtained from the medical image processing packages in [1] are shown in Table 2. The ratio obtained for the ROI section is similar to the latest technology approach in the literature.

Also, the original and the compressed file sizes of the images are shown in Table 3. As the medical images' data size increases, it will be better understood how advantageous the platform is.

### 4.3. Operation Parallelism Results

At this stage of the work, the results of the proposed middle layer platform are presented.

#### 4.3.1. Hadoop/MapReduce

**Figure 12:**
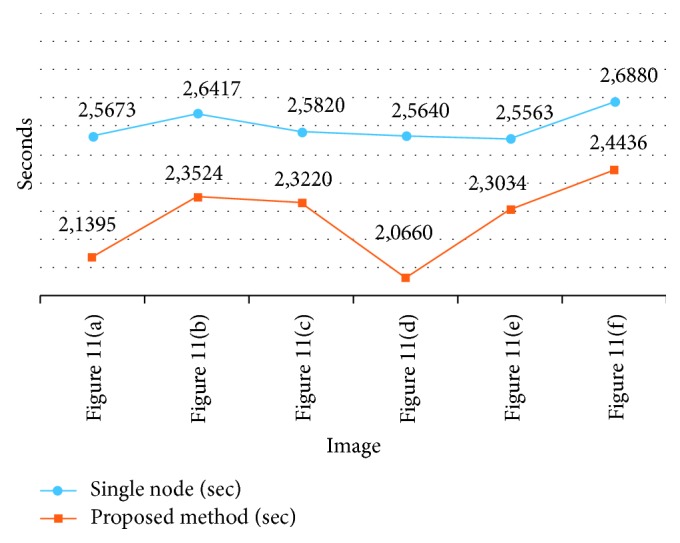
Processing speeds.

The MIPP package proposed in [1] is executed on a single node, but when the number of medical images is considered, performance is required at the deployment stage of the proposed method. The processing speeds of the single node and the MapReduce/Hadoop node of the MIPP are shown in Figure 12.

The data shown above have very close values, but it will be more accurate to compare the total values to evaluate the success of the proposed method as shown in Table 4.

While the single node completes the test images at 15,5993 seconds, the proposed method completes at 8,1537 seconds. If there was only one node in the proposed method, the completion time of the process would be 13,6269 seconds. In the medical image package, *M* images and *N* servers in the cluster are considered: (i) one node performs these operations one by one, and (ii) the proposed method performs *M*/*N* operations. In this respect, the proposed method has been bridging the gap in the literature. Our motivation for using a single node in the experimental results is the current HIMS already using one-node architecture.

#### 4.3.2. MongoDB Performance

The proposed system uses distributed MongoDB, a NoSQL database, for speed, security, and reliability. In the method developed in [1], relational database SQL Server was used. The following query types were used when measuring database speed as shown in Table 5.

To increase the accuracy of the queries, dummy data are added to both databases. The write speeds of the databases have been evaluated in Table 6. According to the results obtained, distributed MongoDB used in the proposed method is faster than SQL Server, which is the relational database, in all registration numbers.

In Table 7, the result is obtained by querying the indexed numerical data. The results show that SQL Server performs well in small amounts of data, so it is worse than MongoDB as the number of records increases. As a result, MongoDB has better results than SQL Server.

Table 8 shows the results of the interrogation with nonindexed fields. According to the performance, MongoDB is performing well in large amounts of data.

As seen in the tables, the higher the number of MongoDB data used in the platform, the higher the performance gain.

## 5. Discussion

Hadoop and MongoDB have been used in the study. Although these tools require expertise during the initial installation phase, they are preferred because of the benefits they provide such as free of license, horizontal growth, and high performance.

Today, HIMS is used in all hospitals, clinics, and health centers. However, these systems do not support multiple thread structures, so they work on a single core. Multinode structures have usually more performance than single-node structures. However, the default settings of these systems are not suitable for multinode construction due to their structures. In this work, a service-based middle layer platform has been developed for the currently used HIMS. The benchmarks in Experimental Results were made between the proposed multinode platform and single-node systems that are currently used. In addition to the performance provided, a new perspective has been provided to the literature.

Thanks to the multinode service-based structure of the developed system, the developed system can be integrated with HIMS at minimal cost and offers an alternative and powerful solution to the archive problem where they are used.

## 6. Conclusion

In this study, an efficient middle layer platform has been developed using the state-of-the-art technologies. A compression with medical image processing package has been implemented using Hadoop/MapReduce, and the obtained data have been stored in sharded MongoDB.

The main contributors to the work done are as follows:

- Medical images have been compressed using the state-of-the-art approach in the literature.
- All the operations have been performed on the Hadoop cluster using the MapReduce programming model.
- The resulting data are stored in a secure sharded MongoDB database.
- The developed fast and efficient search engine provides access to the medical image that the end user searched for safely.
- The developed service-based platform is available to all HIMS for medical imaging archives without changing the DICOM standard.

The work that has been done is bridging the gap in the literature with presented innovations.

## Figures and Tables

**Figure 11:**
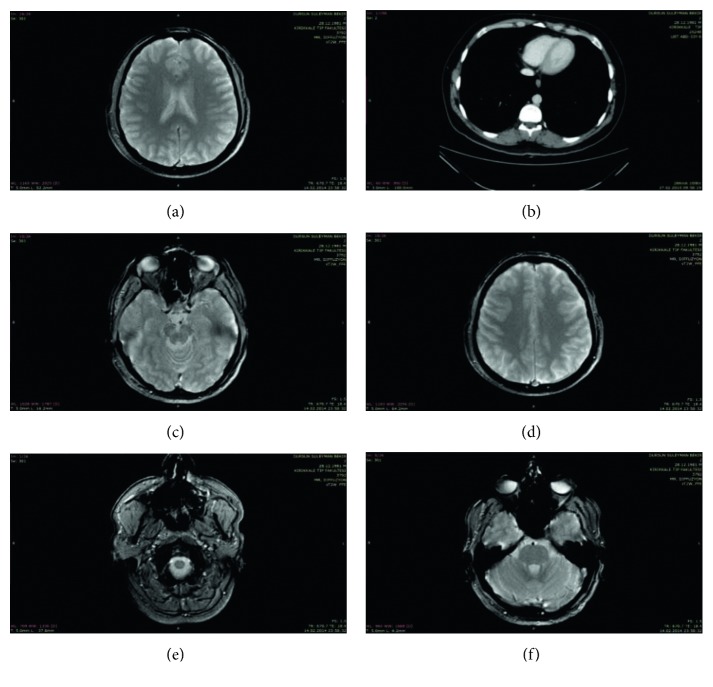
Test medical images.

**Table 1:** Image segmentation performance comparison.

Image	Image segment	Image region (%)	Region-Based file size (MB)	Total file size (MB)
Figure 11(a)	ROI	29.39	2,560	8,712
	Non-ROI	70.61	6,152	

Figure 11(b)	ROI	43.23	3,991	9,231
	Non-ROI	56.77	5,240	

Figure 11(c)	ROI	32.12	2,777	8,647
	Non-ROI	67.88	5,870	

Figure 11(d)	ROI	28.76	2,318	8,059
	Non-ROI	71.24	5,741	

Figure 11(e)	ROI	27.33	2,318	8,482
	Non-ROI	72.67	6,164	

Figure 11(f)	ROI	36.21	3,390	9,361
	Non-ROI	63.79	5,971	

Average	ROI	32.84	2,892	8,749
	Non-ROI	67.16	5,856	

**Table 2:** Segment-based compression ratios.

Image	Image segment	Compression ratio
Figure 11(a)	ROI	3,006
	Non-ROI	92,122

Figure 11(b)	ROI	2,255
	Non-ROI	97,322

Figure 11(c)	ROI	3,916
	Non-ROI	96,451

Figure 11(d)	ROI	3,542
	Non-ROI	95,853

Figure 11(e)	ROI	2,851
	Non-ROI	97,842

Figure 11(f)	ROI	3,246
	Non-ROI	94,739

**Table 3:** File sizes.

Image	Original image file size (MB)	Compressed image file size (MB)
Figure 11(a)	8,712	0,919
Figure 11(b)	9,231	1,823
Figure 11(c)	8,647	0,770
Figure 11(d)	8,059	0,714
Figure 11(e)	8,482	0,876
Figure 11(f)	9,361	1,107
Average	8,749	1,035

**Table 4:** Total time.

	Single node (sec)	Proposed method (sec)
Image average	15,5993	13,6269
Total time	—	8,1537

**Table 5:** Test queries.

Query number	Query description
I	To write data (MIPP data package and indexes)
II	To retrieve results containing one numerical value (“HIMS ID = 52721”)
III	To retrieve results containing search engine criteria (“PatientName = Erdal,” “PatientSurname = Erdal,” “StartDate = 01.01.2016,” “EndData = 01.01.2017”)

**Table 6:** Write data.

Dummy record number	Query 1 response time (MS)	
	SQL server	Sharded MongoDB
1000	1,37	1,28
10,000	14,98	8,11
100,000	143,08	76,28
1,000,000	1409,32	778,94

**Table 7:** Select-indexed data.

Dummy record number	Query 2 response time (MS)	
	SQL server	Sharded MongoDB
1000	1,48	4,83
10,000	6,63	3,56
100,000	33,69	25,72
1,000,000	318,50	276,97

**Table 8:** Select nonindexed data.

Dummy record number	Query 3 response time (MS)	
	SQL server	Sharded MongoDB
1000	3,23	7,56
10,000	13,66	13,95
100,000	79,97	92,44
1,000,000	814,42	650,97
